# Exploring protective and risk factors in the home environment in high-risk families – results from the Danish High Risk and Resilience Study—VIA 7

**DOI:** 10.1186/s12888-022-03733-5

**Published:** 2022-02-09

**Authors:** Anne Amalie Elgaard Thorup, Ditte Lou Gantriis, Aja Neergaard Greve, Maria Toft Henriksen, Kate Kold Zahle, Henriette Stadsgaard, Ditte Ellersgaard, Birgitte Klee Burton, Camilla Jerlang Christiani, Katrine Spang, Nicoline Hemager, Jens Richardt Møllegaard Jepsen, Kerstin J. Plessen, Merete Nordentoft, Ole Mors, Vibeke Bliksted

**Affiliations:** 1 The Lundbeck Foundation Initiative for Integrative Psychiatric Research (iPSYCH), Aarhus, Denmark; 2 Child and Adolescent Mental Health Center, Mental Health Services, Capital Region of Denmark, University of Copenhagen, Copenhagen, Denmark; 3 Department of Clinical Medicine, Faculty of Health and Medical Sciences, University of Copenhagen, Copenhagen, Denmark; 4 Psychosis Research Unit, Aarhus University Hospital Skejby Psychiatry, Aarhus, Denmark; 5 Centre for Neuropsychiatric Schizophrenia Research & Centre for Clinical Intervention and Neuropsychiatric Schizophrenia Research, Copenhagen University Hospital, Copenhagen, Denmark; 6 CORE Copenhagen Research Unit, Mental Health Centre Copenhagen, Copenhagen University Hospital, Mental Health Services Capital Region, Copenhagen, Denmark; 7 Division of Child and Adolescent Psychiatry, Department of Psychiatry, University Medical Center, University of Lausanne, Lausanne, Switzerland

**Keywords:** Home environment, Risk factors, Schizophrenia, Bipolar disorder, Familial high-risk

## Abstract

**Background:**

Exposure to inadequate home environment may put the healthy development of familial high-risk children at risk. This study aimed to investigate associations between risk factors and an adequate home environment of children having a parent diagnosed with schizophrenia or bipolar disorder.

**Methods:**

From a cohort of 522 children, data from 463 7-year-old children was included. Of these 172 children had familial risk for schizophrenia, 109 children had familial risk for bipolar disorder, and 190 were population-based controls. As part of a comprehensive battery, all participants were assessed with the Middle Childhood-Home Observation for Measurement of the Environment Inventory (MC-HOME Inventory) measuring the quality of the home environment.

**Results:**

When analyzing all families together, we found that having a parent diagnosed with schizophrenia would have a negative impact on the home environment (ß = -1.08; 95% CI (-2.16;-0.01); *p* = 0.05), while familial risk for bipolar disorder did not show significant predictive value. Being a single caregiver and child having experienced severe life events from ages 4 to 7 showed significant negative impact, while child having a mental illness diagnosis did not. Being a female caregiver, good social functioning of the caregiver, high child IQ and not being a single caregiver were found to predict positive values for the home environment. We found similar results when analyzing caregivers with and without a diagnosis separately.

**Conclusions:**

Knowledge of what predicts good home environment should be used to inform development of early interventions for families at risk.

**Supplementary Information:**

The online version contains supplementary material available at 10.1186/s12888-022-03733-5.

## Background

A safe and secure home environment should provide a sufficient level of stimulation for the child to explore the surroundings while at the same time giving sensitive support in stressed situations. This will enhance chances of a healthy development [1, 2]. A warm and positive relationship between caregiver and child is also critical for social development [3] and for the development of the sense of a positive self [4]. Thus, positive relations and a supportive and safe home environment also providing the physical necessities like food, clothing and housing are essential for the well-being of a child [5]. Prior studies have shown that children’s cognitive and social development is strongly linked to factors in the home environment [6, 7], and that growing up in an impaired and inadequate home environment poses a risk for social and academic functioning [8, 9], and may result in failure to thrive [10]. Some caregivers have problems providing the relevant and needed support and care for their child due to parental severe mental disorders [11], poverty, lack of knowledge and skills and lack of social support [12, 13]. Further, factors such as living with a single caregiver, having an unemployed parent [7], experiencing unsupportive, neglectful or insensitive parenting [14] are factors in the rearing environment that are also found to pose a risk to the well-being of a child. A Swedish register study documented this increased risk of socioeconomic adversity in children of parents with mental illness compared to well parents [15].

Familial high risk (FHR) studies investigating children of parents with severe mental disorders like schizophrenia and bipolar disorder have found that these children are exposed to a range of environmental risk factors compared to children of parents without mental disorders [16, 17] due to direct and indirect consequences of the mental health problems of the parent. These risk factors have a bidirectional impact on maternal and child wellbeing [16]. Further, children with FHR are at an elevated risk of developing psychopathology [18, 19] that is identifiable already at an early age and display higher rates of neurocognitive deficits [17, 20]. In the Danish High Risk and Resilience study – VIA7, which is a familial high risk study based on a large sample of children (*N* = 522), we found that a larger proportion of the children with a predisposition for schizophrenia also lived in a home environment that was estimated to be of poorer quality than in the population-based controls (PBC) [21].

In summary, being a child with familial high risk for severe mental illness implies a risk of growing up with higher levels of adverse environmental exposures including inadequate stimulation and support in the home environment compared to children whose parents do not have a severe mental disorder. Based on prior research showing that exposure to an inadequate home environment represents a risk to healthy development, we aimed to explore which risk factors pose the greatest risk of an inadequate home environment in a sample of children born to an affected parent. Further, we aimed to investigate if there was a difference in the impact of risk factors between those children whose primary caregiver was the ill parent and those whose primary caregiver was not diagnosed with schizophrenia or bipolar disorder. We hypothesized that risk factors in the family such as single caregiver status, parental unemployment, parental diagnosis of misuse, low level of daily functioning of the parent, low level of intelligence in parent and in child, child diagnosed with a mental illness and high number of severe life events would predict lower levels of stimulation and support in the home environment measured with HOME-MC [22].

## Methods

### Participants

This study reports data from a subsample of the nationwide Danish High Risk and Resilience Study – VIA7 (hereafter referred to as the VIA7 study). The VIA7 study was conducted in Denmark from January 1, 2013 to January 31, 2016. The overall study design of the VIA7 study has been described in detail elsewhere [23]. Subjects were identified through the Danish Civil Registration System and the Danish Psychiatric Central Research register. Schizophrenia spectrum psychosis (SZ) was defined as delusional disorder and schizoaffective disorder (ICD 10-codes: F20, F22 and F25 or ICD 8-codes: 295, 297, 298.29, 298.39, 298.89, 298.99). Bipolar disorder (BP) was defined by ICD 10 code F30, F31 or ICD 8-codes: 296.19, 296.39). A list of 10 controls was drawn up for each ‘case’, and the best match as to gender, municipality and exact age was aimed for. The control children were defined as being born to parents who had never been diagnosed with schizophrenia or bipolar disorder, but who could have or have had other kinds of mental health problems. An exclusion criterion for the study was adoption.

We chose age seven since we assumed that at that time the mother/primary caregiver would still be able to remember the early years of the child’s life and development in an anamnestic interview.

At age seven all children would have started school, which is a developmental milestone where demands on the child increase in terms of ability to pay sustained attention, receive collective information, navigate with peers, handle practical issues alone etc. The subsample used for this paper was data from families where the primary caregiver took part in the MC-HOME Inventory (*N* = 487), the relevant instrument for the purpose of this work. The primary caregiver was defined as the parent or foster parent who knew the child the best and spent most time with the child, and who was identified in collaboration with the parents. All meetings with the family were arranged in a flexible manner to fit with their other obligations (work, school, etc.), even outside office hours if needed. We also assisted participating families in overcoming any practical obstacles like transportation etc. In all families, two testers were assigned, the one who tested the child was kept blinded for the high risk status of the family during the entire testing period. Furthermore, in case of siblings (*N* = 16), parent and child information was only included from the first included sibling in order not to count the same parent twice. Thus, this study sample ends up consisting of 471 7-year-old children having a parent diagnosed with SZ (*N* = 172) or BP (*N* = 109) and population based controls (PBC) (*N* = 190) who did not have a diagnosis of SZ or BP. Due to missing values in the predictors used in our analyses we ended up with *N* = 463. For example, we had missing data on three children's IQ, missing data on two children’s diagnosis and missing data on five children on parental IQ and parental functioning. These were all missing at random among the three groups and were due to e.g. fatigue or not having sufficient time for completing the last tests.

### Procedure

After receiving verbal and written information about the study, all the participating adults (or legal guardians of the child) gave written informed consent. The study was approved by the Danish Data Protection Agency. The Capital Region of Denmark’s Committee on Health Research Ethics reviewed the protocol for approval, however, due to the absence of intervention, ethical approval was not deemed necessary. Assessment of the families was mainly conducted at the Psychosis Research Unit, Aarhus University Hospital Risskov, at the Research Unit, Mental Health Centre Copenhagen and in the homes of the participating families. The assessors were nurses, psychologists and medical doctors trained and supervised by certified clinical specialists. Child assessors were blinded to the diagnoses of the parents.

### Measures

#### Home environment

The home environment was measured with the Middle Childhood HOME Inventory (MC-HOME Inventory) for children aged 6–10, also known as the Elementary HOME [22]. The MC-HOME Inventory is designed to identify potentially inadequate home environments that could pose a risk to a child´s development [22, 24]. It consists of eight subscales, each consisting of 4–10 items, that measure different aspects of stimulation and support available in the home environment. Examples of subscales are the Emotional climate subscale measuring the caregiver´s acceptance of negative emotional expressions and the emotional self-control of the caregiver. Another subscale measures the availability of learning materials and opportunities available for the child. The subscale of Enrichment captures the extent to which the family utilizes community resources to support the development of the child in terms of hobbies, recreational activities, trips etc., while another subscale measures the physical environment^22^(Table 1S). The interview must always take place in the home of the child and requires that the assessor interacts with the child and the caregiver. The assessor was blinded for familial high risk status and followed a manual with an outline of how to inquire about or observe the content of the 59 items of the interview that were scored on site and how different answers should be interpreted and scored. Any doubts were always discussed with a colleague, and often two researchers were present in the home. Duration is approximately 45–60 min. No specific cut-off scores are defined of a risk environment, but the primary inventor of the instrument suggests that a score below 1–2 standard deviations of the mean of a healthy population should be interpreted as a potentially inadequate home environment [22]. The cut-off is dependent on culture [25].

#### Training and inter-rater reliability

A Danish MC-HOME Inventory administration manual was used to assess the quality of the home environment. The development of the Danish manual followed the method of the adaption process described by Beaton (2000) [26]. One of the developers of the MC-HOME Inventory, Professor R. Bradley, approved of the translation (Bradley, personal communication). The training of the MC-HOME Inventory interview consisted of a 6 hour introduction including reading the HOME Inventory Administration Manual, watching videos of trained interviewers conducting MC-HOME Inventory interviews and discussing the method. All assessors performed at least two “practice” interviews not part of the VIA7 study data collection, and assessors received supervision from the first and second authors (DLG, AT) throughout the data collection period. Inter-rater reliability was assessed using Intra-class Correlation Coefficient analysis comparing the variability of 11 independent ratings of the same 10 subjects to the total variance across all ratings and all subjects. The Intra-class Correlation Coefficient for the total score was 0.98 (95% CI: 0.96–1.00) on average score and 0.83 (95% CI: 0.68-0.94) on the individual score.

#### Clinical and demographic measures

Information about employment, number of experienced severe life events (e.g. death of a close relative, accidents or somatic illness, divorce or serious problems in partner relationship, problems with work or unemployment, problems with housing or financial circumstances), and the single parent status of the person was obtained through structured anamnestic interviews with the primary caregivers. Employment was defined as being in employment (including temporary leave) or following a recognized educational program for a minimum of 15 h weekly.

Level of intelligence of parents and children was assessed with the Reynolds Intellectual Screening Test (RIST) derived from the Reynolds Intellectual Assessment Scale (RIAS) [27]. Present diagnoses of the children were identified from the semi-structured psychopathology screening interview Kiddie Schedule for Affective Disorders and Schizophrenia, Present and Lifetime version; K-SADS-PL [28].

Any life time diagnosis of substance use of the caregivers was registered as part of the psychopathology screening interview (SCAN, Schedules for Clinical Assessment in Neuropsychiatry) following ICD-10 [29]. Social functioning of the caregiver was measured with PSP (Personal and Social Performance Scale [30]).

#### Data analyses

Only primary caregivers with a full MC-HOME Inventory score and only the first included siblings were included in the analyses. We used Chi-square test for dichotomous variables and one-way analyses of variance (ANOVA) for continuous variables. Prior to statistical analyses the data was inspected and tested for normality. We chose not to adjust for IQ, education or unemployment in order not to over-adjust group differences that may be due to the possibly same underlying biological and genetical factors influencing the outcome measures [31].

The home environment was evaluated mainly on the basis of the previous month’s events. We deliberately chose the content of our model based on factors that were present for a longer period of time than the current month and thus could be considered as potential predictors. In order to assess the association between various predictors from the primary caregiver and child and the MC-HOME Inventory total score multiple linear regression models were applied. Furthermore, we made a supplementary multiple logistic regression analysis to assess the association between a number of important predictors and having a poor home environment defined as a MC-HOME Inventory total score ≤ 40 [21]. The statistical analyses were performed using the statistical software Stata version 16.0.

## Results

### Demographics

The gender distribution was similar among the three groups of children. The estimated IQ of the FHR-SZ children was slightly lower than that of the other children and differed significantly from the PBC children (FHR-SZ: m = 102.58 sd = 11.10; FHR-BP: m = 104.08 sd = 9.42; PBC: m = 105 sd = 10.07; FHR-SZ versus PBC: *p* = 0.03). The familial high risk children had had more severe life events overall than the PBC children. The gender distribution of the primary caregivers did not differ among the three groups. However, the FHR-SZ caregivers were younger and had a lower estimated IQ than the other primary caregivers. More primary caregivers were single in the two familial high risk groups compared to the PBC. The primary caregivers of both familial high risk groups had lower level of functioning, more were often unemployed and more had a diagnosis of substance use (Table 1).

**Table 1:** Sample characteristics of children and primary caregivers

	**N**	**FHR-SZ**	**FHR-BP**	**PBC**	*P*-value^f^	*P*-value^f^ Pairwise comparison		
						**FHR-SZ vs. PBC**	**FHR-BP vs. PBC**	**FHR-BP vs. FHR-SZ**
**Children**^**a**^	471	172	109	190				
Females, N (%)	223/471	85 (49.42)	52 (47.71)	86 (45.26)	0.73	0.43	0.68	0.78
Children's IQ^b^, mean (SD)	468	102.58 (11.10)	104.08 (9.42)	105 (10.07)	0.08	**0.03**	0.44	0.24
Severe life events 0–3 years old	471				** < 0.001**	** < 0.001**	** < 0.001**	0.48
0		63 (36.63)	48 (44.04)	114 60.00)				
1		56 (32.56)	28 (25.69)	55 (28.95)				
2		40 (23.26)	27 (24.77)	15 (7.89)				
3		13 (7.56)	6 (5.50)	6 (3.16)				
Severe life events 4–7 years old:	471				**0.004**	**0.001**	**0.023**	0.60
0		45 (26.16)	32 (29.36)	78 (41.05)				
1		52 (30.23)	33 (30.28)	65 (34.21)				
2		50 (29.07)	34 (31.19)	32 (16.84)				
3		25 (14.53)	10 (9.17)	15 (7.89)				
K-SADS-PL^c^ diagnosis present	469				**0.002**	**0.001**	**0.012**	0.67
No		97 (57.06)	65 (59.63)	140 (73.68)				
Yes		73 (42.94)	44 (40.37)	50 (26.32)				
**Primary caregivers**^**d**^	471	172	109	190				
Female primary caregivers, N (%)	430/471	155 (90.12)	102 (93.58)	173 (91.05)	0.60	0.76	0.44	0.31
Primary caregiver's age, mean (SD)	471	37.50 (5.95)	39.41 (5.71)	40.06 (4.15)	** < 0.001**	** < 0.0001**	0.26	**0.01**
Primary caregiver´s IQ^b^, mean (SD)	466	102.43 (8.80)	104.94 (8.23)	103.97 (7.96)	**0.04**	0.08	0.32	**0.02**
Primary caregiver is single, N (%)	123/471	68 (39.53)	34 (31.19)	21 (11.05)	** < 0.001**	** < 0.001**	** < 0.001**	0.16
Primary caregiver´s level of functioning^e^, mean (SD)	466	72.71 (14.28)	74.70 (14.08)	84.38 (9.20)	** < 0.0001**	** < 0.001**	** < 0.0001**	0.26
Primary caregiver is unemployed	355/471	62 (36.05)	36 (33.03)	18 (9.47)	** < 0.001**	** < 0.001**	** < 0.001**	0.61
Diagnosis of substance use	22/471	13 (7.56)	9 (8.26)	0 (0)	** < 0.001**	** < 0.001**	** < 0.001**	0.83

^a^In the case of siblings, parent and child information is only included from the first included sibling in order not to count the same parent twice. ^b^Reynolds Intellectual Screening Test (RIST). ^c^KSADS-PL, Kiddie Schedule for Affective Disorders and Schizophrenia. ^d^Primary caregiver is defined as the parent or forster parent that knows the child the best and spends most time with the child. ^e^Personal and Social Performance Scale (PSP).^f^Data was analysed with either one-way analyses of variance (ANOVA) (continuous variables) or Chi-squared test (categorical variables)

### Multiple regression of potential predictors in the total sample

In step one we investigated potential predictors of increasing MC-HOME Inventory scores to learn what contributes to a good home environment among children and primary caregivers (*N* = 463 families) (Table 2).

**Table 2:** Prediction of a good home environment as measured by the MC-HOME Inventory total score based on data from the children and the primary caregivers^.^ Primary caregiver is defined as the parent or foster parent that knows the child best and spends most time with the child^a^. (*N* = 463)

Predictors	ß	SE	t	*p*-value	95%CI
**Intercept**	16.64	3.90	4.27	** < 0.001**	(8.98;24.31)
**Caregiver’s age**	0.04	0.04	1.05	0.30	(-0.04;0.13)
**Child’s sex**					
Male	0				
Female	-0.48	0.43	-1.11	0.27	(-1.31;0.36)
**Caregiver’s sex**					
Male	0				
Female	1.68	0.76	2.21	**0.03**	(0.19;3.17)
**IQ child**^**b**^	0.10	0.02	4.42	** < 0.001**	(0.05;0.14)
**IQ caregiver**^**b**^	0.06	0.03	2.31	**0.02**	(0.01;0.12)
**Caregiver single**	0				
**Caregiver not single**	2.25	0.51	4.40	** < 0.001**	(1.25;3.26)
**FHR**^c^					
FHR-SZ	-1.08	0.55	-1.98	**0.05**	(-2.16;-0.01)
FHR-_BP	-0.58	0.59	-0.98	0.33	(-1.74;0.58)
PBC	0				
**Caregiver unemployed**	0.82	0.67	1.22	0.22	(-0.50;2.14)
**Caregiver employed**	0				
**Severe life events, 0–3 years old**					
0	0				
1	0.34	0.51	0.66	0.51	(-0.67;1.34)
2	0.03	0.65	0.04	0.97	(-1.25;1.30)
3	0.59	1.02	0.57	0.57	(-1.43;2.60)
**Severe life events 4–7 years old**					
0	0				
1	-0.07	0.53	-0.12	0.90	(-1.12;0.98)
2	-1.41	0.61	-2.32	**0.02**	(-2.61;-0.22)
3	-1.80	0.81	-2.22	**0.03**	(-3.39;-0.21)
**K-SADS-PL**^**d**^** present diagnosis**					
No	0				
Yes	-0.49	0.46	-1.05	0.29	(-1.40;0.42)
**Diagnosis of substance use of primary caregiver**					
No	0				
Yes	0.15	1.03	0.15	0.88	(-1.87;2.18)
**PSP primary caregiver**	0.13	0.02	5.54	** < 0.001**	(0.08;0.17)
	F(18,444) = 12.76; *p* < 0.0001; R^2^ = 0.34; Adj. R^2^ = 0.31; Root MSE = 4.50				

^a^In the case of siblings; parent and child information is only included from the first included sibling in order not to count the same parent twice. ^b^*RIST* Reynolds Intellectual Screening Test. ^c^Familiar High Risk status (*SZ* Schizophrenia, *BP* Bipolar disorder, *PBC* population based controls). ^d^*K-SADS-PL* Kiddie Schedule for Affective Disorders and Schizophrenia

Regarding the primary caregiver, age, employment status, a diagnosis of substance use and number of severe life events when the child was 0–3 years did not predict the quality of the home environment as measured by MC-HOME Inventory Caregivers being female (ß = 1.68; 95% CI (0.19;3.17); *p* = 0.03), not being single (ß = 2.25; 95% CI (1.25;3.26); *p* < 0.001) and higher level of IQ of the adult (ß = 0.06; 95% CI (0.01;0.12); *p* = 0.02) and adult level of social functioning (ß = 0.13; 95% CI (0.08;0.17); *p* < 0.001) were found to have a positive impact on the home environment score, e.g. better social functioning, for instance, predicting a better home environment. Familial high risk of bipolar disorder (FHR-BP) was not found to impact the home environment, while FHR-SZ was seen to have a negative predictive value on the level of the MC-HOME Inventory score (ß = -1.08; 95% CI (-2.16;-0.01); *p* = 0.05).

We found that having experienced more than one severe life event between age 4 and 7 had a negative impact on the home environment (two events: ß = -1.41; 95% CI (-2.61;-0.22); *p* = 0.02, three events: ß = -1.80; 95% CI (-3.39;-0.21); *p* = 0.03). The sex of the child or whether the child had a current psychiatric diagnosis did not seem to influence the home environment score, while higher IQ of the child predicted a better home environment (ß = 0.10; 95% CI (0.05;0.14); *p* < 0.001).

We also investigated the potential predictors of a good home environment defined as a MC-MC-HOME Inventory cutoff score above 40 [21] (*N* = 463 families, Table 2S). The primary caregiver not being single had a positive influence on the home environment (ß = 0.07; 95% CI (0.01;0.14); *p* = 0.03) as did the social functioning of the primary caregiver (ß = 0.006; 95% CI (0.003;0.009); *p* < 0.001). However, having a diagnosis of SZ had a negative impact (ß = -0.08; 95% CI (-0.15; -0.01); *p* = 0.04). Furthermore, a higher IQ of the child had a positive impact on the home environment (ß = 0.005; 95% CI (0.002;0.008); *p* < 0.001).

### Multiple regression of potential predictors in the index primary caregivers

In the second step we only used data from those families where the primary caregiver was also the index parent (*N* = 253 families), i.e. the parents who were diagnosed with either bipolar disorder or schizophrenia or their adult matched population based control subject (Table 3).

**Table 3:** Prediction of a good home environment as measured by the MC-HOME Inventory total score based on data from the primary caregivers who are index parents and their children. An index parent is defined as the parent with a diagnosis of either schizophrenia spectrum disorder or bipolar disorder or their adult matched population-based control subject^a^. (*N* = 253)

Predictors	ß	SE	t	*p*-value	95%CI
**Intercept**	21.40	5.16	4.15	** < 0.001**	(11.25;31.56)
**Caregiver’s age**	0.07	0.06	1.27	0.21	(-0.04;0.18)
**Child’s sex**					
Male	0				
Female	-0.22	0.56	-0.40	0.69	(-1.32;0.88)
**Caregiver’s sex**					
Male	0				
Female	1.02	1.20	0.85	0.40	(-1.34;3.37)
**IQ child**^**b**^	0.08	0.03	2.48	**0.01**	(0.02;0.14)
**IQ caregiver**^b^	0.04	0.04	1.16	0.25	(-0.03;0.11)
**Caregiver single**	0				
**Caregiver not single**	3.00	0.71	4.23	** < 0.001**	(1.60;4.39)
**FHR**^c^					
FHR-SZ	0.09	0.74	0.13	0.90	(-1.37;1.55)
FHR-BP	0.45	0.79	0.57	0.57	(-1.11;2.01)
PBC	0				
**Caregiver unemployed**	0.74	0.82	0.91	0.34	(-0.87;2.36)
**Caregiver employed**	0				
**Severe life events 0–3 years old**					
0	0				
1	0.64	0.65	0.99	0.32	(-0.64;1.92)
2	0.49	0.84	0.59	0.56	(-1.16;2.15)
3	1.45	1.35	1.07	0.29	(-1.22;4.11)
**Severe life events 4–7 years old**					
0	0				
1	-1.13	0.72	-1.56	0.12	(-2.56;0.29)
2	-2.14	0.75	-2.86	**0.005**	(-3.63;-0.66)
3	-2.43	1.00	-2.43	**0.02**	(-4.41;0.46)
**K-SADS-PL**^d^ **present diagnosis**					
No	0				
Yes	-0.71	0.61	-1.16	0.25	(-1.90;0.49)
**Diagnosis of substance use of primary caregiver**					
No	0				
Yes	-1.33	1.20	-1.11	0.27	(-3.70;1.04)
**PSP primary caregiver**	0.11	0.03	3.76	** < 0.001**	(0.05;0.17)
	F(18,234) = 6.45; *p* < 0.0001; R^2^ = 0.33; Adj. R^2^ = 0.28; Root MSE = 4.27				

^a^In the case of siblings; parent and child information is only included from the first included sibling in order not to count the same parent twice. ^b^*RIST* Reynolds Intellectual Screening Test. ^c^Familiar High Risk status (*SZ* Schizophrenia, *BP* Bipolar disorder, *PBC* population-based controls). ^d^*K-SADS-PL*, Kiddie Schedule for Affective Disorders and Schizophrenia

Regarding the families where the index parent (i.e. the parent with a diagnosis) was also the child’s primary caregiver, age, sex, IQ, familiar risk status, employment, lifetime diagnosis of substance use and number of severe life events during age 0–3 of the child were not found to impact the home environment. However, we did find that not being single (ß = 3.00; 95% CI (1.60;4.39); *p* < 0.001) and having a high level of social functioning (ß = 0.11; 95% CI (0.05;0.17); *p* < 0.001) had a positive impact on the home environment (e.g. higher social functioning predicted a better home environment) when the index parent was the caregiver. On the other hand, having experienced two or more severe life events when the child was 4 to 7 years old had a negative impact on the quality of the home environment (Two events: ß = -2.14; 95% CI (-3.63; -0.66); *p* = 0.005. Three events: ß = -2.43; 95% CI (-4.41;0.46); *p* = 0.02). The sex of the child and whether the child had a mental health diagnosis or not did not influence the home environment. The child’s IQ was positively correlated with a good home environment (e.g. higher IQ was associated with a better home environment) (ß = 0.08; 95% CI (0.02;0.14); *p* = 0.01).

### Multiple regression of potential predictors of the non-index primary caregivers

In step three we analyzed only data from the families where the primary caregiver was the non-index parent, i.e. the caregiver was not the parent diagnosed with SZ or BP (*N* = 210 families) (Table 4).

**Table 4:** Prediction of a good home environment as measured by the MC-HOME Inventory total score based on data from the primary caregivers who are not index parents. An index parent is defined as the parent with a diagnosis of either schizophrenia spectrum disorder or bipolar disorder or their adult matched population-based control subject^a^. (*N* = 210)

Predictors	ß	SE	t	*p*-value	95%CI
**Intercept**	10.50	6.10	1.72	0.09	(-1.54;22.55)
**Caregiver’s age**	0.07	0.06	1.05	0.30	(-0.06;0.19)
**Child’s sex**					
Male	0				
Female	-0.67	0.66	-1.04	0.30	(-1.99;0.62)
**Caregiver’s sex**					
Male	0				
Female	2.21	1.06	2.09	**0.04**	(0.13;4.29)
**IQ child**^**b**^	0.09	0.03	2.79	**0.006**	(0.03;0.15)
**IQ caregiver**^b^	0.06	0.04	1.46	0.15	(-0.02;0.15)
**Caregiver single**	0				
**Caregiver not single**	1.33	0.76	1.75	0.08	(-0.17;2.84)
**FHR**^c^					
FHR-SZ	-1.80	0.88	-2.05	**0.04**	(-3.53;-0.07)
FHR-BP	-2.27	0.92	-2.46	**0.02**	(-4.09;-0.45)
PBC	0				
**Caregiver unemployed**	0.002	1.15	< 0.01	0.998	(-2.27;2.28)
**Caregiver employed**	0				
**Severe life events 0–3 years old**					
0	0				
1	-0.26	0.82	-0.32	0.75	(-1.88;1.36)
2	-0.22	1.04	-0.21	0.83	(-2.27;1.82)
3	-0.24	1.59	-0.15	0.88	(-3.37;2.89)
**Severe life events 4–7 years old**					
0	0				
1	1.11	0.80	1.38	0.17	(-0.47;2.68)
2	-0.05	1.03	-0.05	0.96	(-2.08;1.98)
3	-0.82	1.44	-0.57	0.57	(-3.67;2.03)
**K-SADS-PL**^d^ **present diagnosis**					
No	0				
Yes	-0.38	0.74	-0.51	0.63	(-1.84;1.09)
**Diagnosis of substance use of primary caregiver**					
No	0				
Yes	3.37	1.96	1.72	0.09	(-0.50;7.23)
**PSP primary caregiver**	0.20	0.04	5.02	** < 0.001**	(0.12;0.28)
	F(18,191) = 8.05; *p* < 0.0001; R^2^ = 0.43; Adj. R^2^ = 0.38; Root MSE = 4.61				

^a^In the case of siblings; parent and child information is only included from the first included sibling in order not to count the same parent twice. ^b^*RIST* Reynolds Intellectual Screening Test. ^c^Familiar High Risk status (*SZ* Schizophrenia, *BP* Bipolar disorder, *PBC* population-based controls). ^d^*K-SADS-PL*, Kiddie Schedule for Affective Disorders and Schizophrenia

Regarding the primary caregivers, age, IQ, not being single, employment status, substance use and severe life events when the child was 0–3 years old did not impact the home environment. Being a female caregiver (ß = 2.21; 95% CI (0.13;4.29); *p* = 0.04) had a positive effect on the home environment. Being a primary caregiver with a co-parent with either SZ or BP had a negative impact on the home environment (SZ: ß = -1.80; 95% CI (-3.53;-0.07); *p* = 0.04. BP: ß = -2.27; 95% CI (-4.09;-0.45); *p* = 0.02). The gender of the child and whether the child was diagnosed with a mental disorder or not did not impact the home environment. The IQ of the child had a positive impact on the home environment (e.g. higher IQ was associated with a better home environment) (ß = 0.09; 95% CI (0.03;0.15); *p* = 0.006).

## Discussion

In a large familial high risk cohort of 522 7-year old children, where the majority of them had a parent diagnosed with either schizophrenia or bipolar disorder, we investigated predictors of a good home environment. We found that being a female caregiver, cohabiting parents and good adult social functioning positively influenced the quality of the home environment, measured by the MC-HOME Inventory. Familial predisposition for schizophrenia and child having experienced several severe life events had a negative impact on the home environment. These results did not change much when analyses were broken down into those families where the primary caregiver was also the parent with a diagnosis of SMI and those where the caregiver was ‘the other/well’ parent. Among child characteristics we found that higher child IQ predicted better home environment scores, while mental illness of the child was insignificant.

Assessing the place where the child lives, acts and learns is a powerful tool when trying to capture the important aspects of a child’s developmental processes, since parenting and daily environment are highly influential [32]. Thus, it also represents a potential for prevention and early intervention in families with many risk factors. The HOME Inventory is a validated instrument that has proven its value by being widely used and also found reliable and useful in very different cultural contexts with few adaptations [33]. It measures in an objective manner indicators of what theory points at as being crucial experiences in the child’s home that would promote and support well-being and healthy development. It thus mainly contains issues that are thought to be causal indicators of what is good for the child’s development, always focusing on the child as a recipient of inputs from the surroundings [33]. Although MC-HOME Inventory is a continuous, linear measure, sensitivity is not the same throughout the scale, since the scale is more sensitive at the level that indicates if the environment is sufficient or not, compared to the upper end, where good and superior environments may not be separated. Our analyses were based on a continuous linear assumption and should thus be interpreted with this in mind. In practical terms, it has a larger effect on child development to increase the lower home scores than to increase the higher ones. Our analyses only give information about which elements were found influential. This means that the MC-HOME Inventory is a measure of indices rather than a scale [33].

In this study we aimed to assess the impact of some of the known risk factors that have already been proposed to be associated with poorer family functioning and other negative childhood circumstances. One factor that was investigated is the single parent status, which is quite common in Denmark. It has been shown that single parenting can be associated with less stimulation and poorer material resources and could thus potentially be disadvantageous for a child. This is a logical consequence as two adults can provide more stimulation and support than one, but at the same time it is important to point out that a single parent can do very well as a parent, even if it takes an extra effort. In the MC-HOME inventory the opportunities for higher scores are more prevalent if two adults live together and can provide the issues investigated, in fact, one item directly reports ‘single caregiver in household’. The PSP-score is an estimate of the level of the caregiver’s social and personal functioning in the previous month and was found to be highly correlated with the MC-HOME Inventory score in all three analyses. This is meaningful since many of the measured items strongly rely on the parent/caregiver ability to take the responsibility for the provision of a relevant level of stimulation, structure, routines and support, encouraging the child’s independence in daily life, providing material goods for hobbies and activities and offering warmth and sensitive caring – all aspects of parenting that can be adversely affected by the consequences of severe mental illness. From a preventive perspective, supporting the parent’s daily functioning, e.g. by offering parental training or personal support, would most likely be effective also in terms of improving the home environment for the child. Further, initiatives that strengthen the social support through mapping the social network and encouraging social contacts for the family could be another approach. This is one of the focus points, among others, of one of the most used and evidence based interventions, Beardslee’s Family Talk [34]. There are however no national guidelines or systematic early interventions for these children or their families in Denmark yet.

A child’s mental illness diagnosis did not seem to influence the MC-HOME Inventory score, which could be an indication that the parents manage to adapt to the child’s special needs and still provide a good home environment. For instance, having a clear daily structure and fixed rules about homework and bedtime is helpful for most children, also those with a diagnosis. This also shows that a good environment can be provided in many ways, and if the child does not like to go out on excursions or see live concerts (items from MC-HOME Inventory) then positive scores can be achieved in other ways. It could also be speculated if externalizing compared to internalizing child disorders would have a different impact on the home environment, but this was out of scope of the current study.

We also saw that there were only minor differences between the subgroup where the caregiver was also the one with a diagnosis and the subgroup where the caregiver is not the ill person. This could indicate that if the person with the diagnosis is also the caregiver, this means that he/she is quite well-functioning – or that the impact of the mental illness is the same, no matter who has the diagnosis. It is important to remember that some parents were at full remission or had only minor symptoms, while others were more influenced by their mental health problems. In other words, the way mental illness can affect family life and interpersonal relations varies a great deal, also from a more psychological perspective. Many children and parents experience feelings of loss and sorrow in relation to having to deal with mental illness, either their own or the other parent’s [35].

Strengths: This study has the strength of being the first study to assess the association between known exposures and risk factors and associations with the quality of the home environment in a large sample with a narrow age range. Only a minor proportion of the participating families did not provide sufficient data for the interview to be included in the analysis. The high participation rate is most likely due to the flexible and friendly approach of the assessors. The substantial inter-rater reliability [36] indicates that with a sufficient amount of traning different assessors can obtain similar results using the MC-HOME Inventory. The raters were blinded to the familial risk status, which minimizes the risk of assessor bias. The R2 values for the regression models were good, indicating that the models explained a reasonably high amount of variance.

Limitations: In Denmark, it is not unusual for some children to live with separated parents by dividing their time equally between the two parents’ homes – but we only measured one home environment, namely where the child’s address was registered/the primary caregiver. Some positive things for a child’s development are not part of the MC-HOME Inventory, e.g. having a pet, which some families mentioned as important for their child. General limitations of the study are described elsewhere [23]. Results should be interpretated with caution since some of the risk factors investigated may be partly overlapping, e.g. parental mental illness and risk of experiencing severe life events (Brandt, J. submitted).

## Conclusion

By exploring exposure to risk factors and their association with decreasing scores of the quality of the home environment of children with FHR, this study contributes to the understanding of what influences the home environment and thus also our opportunities for acting and supporting this high-risk population. For example, adult mental health services could pay more attention to the role of parenting of their patients and support their recovery process also in terms of increasing daily functioning in the home, since this will have direct impact on the children according to our resutls. Our hope is that the results from this study will also increase the awareness of possible interventions regarding the quality of stimulation and support children with FHR to help prevent these children from developing behavioral difficulties and psychopathology. One option is to provide parental or family support by offering parental training and at the same time implementing initiatives that reduce risk of severe life events and trauma in these children’s lives. Our research also encourages a more family-based and holistic approach to patients in families where a parent has a mental illness to supplement treatment, e.g. from adult psychiatry.

### Significant outcomes

- Predictors of a good home environment were analyzed in a large familial high-risk cohort of 522 7-year old children, the majority of whom had a parent diagnosed with either schizophrenia or bipolar disorder based on data from home visits (MC-HOME Inventory)
- Being a female caregiver, cohabiting parents and good adult social functioning positively influenced the quality of the home environment. Higher child IQ predicted better home environment scores, while mental illness of the child was insignificant
- Familial predisposition for schizophrenia and child having experienced several severe life events had a negative impact on the home environment, and results did not change much when analyses were broken down into those families where the caregiver was also the parent with a diagnosis of SMI and those where the caregiver was ‘the other/well’ parent
- Results should be used for developing early interventions for improving the home environment for families at risk

### Limitations

- The full cohort consists of 522 families, but some did not participate in the home environment assessment, resulting in only 463 families providing data for this work
- The MC-HOME Inventory is a continouos scale, but more sensitive at the lower end (cut-off) and results should be interpretated with caution
- Children with two homes are only assessed in one of them

## Supplementary Information

**Additional file 1: Table 1S. **Overview of subscales of MC-HOME and risk factors of the rearing environment.

**Additional file 2: Table 2S.** Prediction of a good home environment as measured by the MC-HOME cutoff score (total score <40) based on data from the children and the primary caregivers^.^ Primary caregiver is defined as the parent or foster parent that knows the child best and spends most time with the child^1^. (N=463).

## Data Availability

The correspondig author had full access to all data in the study and had final responsibility for the decision to submit for publication. The datasets used and analysed during the current study are available from the corresponding author on reasonable request.
