# Supplementary material for: Exploring protective and risk factors in the home environment in high-risk families – results from the Danish High Risk and Resilience Study—VIA 7
Source: BMC Psychiatry. 2022 Feb 9;22:100. doi: 10.1186/s12888-022-03733-5 (PMC8827286; doi:10.1186/s12888-022-03733-5)
Supplement: Supplementary file 1 — Additional file 1: Table 1S. Overview of subscales of MC-HOME and risk factors of the rearing environment. [file 12888_2022_3733_MOESM1_ESM.docx]

| **Table 1S Overview of subscales of MC-HOME and risk factors of the rearing environment** | | |
| --- | --- | --- |
| **The eight subscales of the MC-HOME Inventory** | **Items of the MC-HOME Inventory (Scored with a + or -)** | **Risk factors of caregivers** |
| 1. RESPONSIVITY | Parent sometimes yields to child’s fears or rituals (allows night light, accompanies child to new experiences, etc). | Parental mental disorder, unsupportive, insensitive, unresponsive caregiving, negative communication style, stress. |
| 1. ENCOURAGEMENT | Parent is consistent in establishing or applying family rules. | Parental mental disorder, unavailable, insensitive, unsupportive caregiving. |
| 1. EMOTIONAL CLIMATE | Child can express negative feelings toward parents without harsh reprisals | Neglectful and insensitive adult-child interaction, flat and incongruent affect, delusions, insensitive and unavailable caregiving, stress, maltreatment including spanking |
| 1. LEANING MATERIALS & OPPORTUNITIES | Child has free access to musical instrument (piano, drum, ukulele, or guitar, etc.). | Unsupportive caregiving, stress, parental mental disorder. |
| 1. ENRICHMENT | Family encourages child to develop or sustain hobbies | Flat affect, unsupportive caregiving, parental mental disorder. |
| 1. FAMILY COMPANIONSHIP | Parent helps child to achieve advance motor skills – ride a two-wheel bicycle, roller skate, ice skate, play ball, etc. | Unavailability, flat and incongruent affect, delusions, insensitivity, stress, parental mental disorder. |
| 1. FAMILY INTEGRATION | Child eats at least 1 meal per day, on most days, with mother and father (or mother and father figures). | Parental mental disorder. |
| 1. PHYSICAL ENVIRONMENT | The building has no potentially dangerous structural or health defects. | Poverty. |
