# Supplementary material for: Exploring protective and risk factors in the home environment in high-risk families – results from the Danish High Risk and Resilience Study—VIA 7
Source: BMC Psychiatry. 2022 Feb 9;22:100. doi: 10.1186/s12888-022-03733-5 (PMC8827286; doi:10.1186/s12888-022-03733-5)
Supplement: Supplementary file 2 — Additional file 2: Table 2S. Prediction of a good home environment as measured by the MC-HOME cutoff score (total score <40) based on data from the children and the primary caregivers. Primary caregiver is defined as the parent or foster parent that knows the child best and spends most time with the child1. (N=463). [file 12888_2022_3733_MOESM2_ESM.docx]

**Table 2S.** Prediction of a good home environment as measured by the MC-HOME cutoff score (total score <40) based on data from the children and the primary caregivers^.^ Primary caregiver is defined as the parent or foster parent that knows the child best and spends most time with the child^1^. (N=463).

| **Predictors** | **ß** | **SE** | **t** | **p-value** | **95%CI** |
| --- | --- | --- | --- | --- | --- |
| **Intercept** | -0.42 | 0.26 | -1.60 | 0.11 | (-0.94;0.10) |
| **Caregiver’s age** | 0.0002 | 0.003 | 0.06 | 0.96 | (-0.005;0.006) |
| **Child’s sex** |  |  |  |  |  |
| Male | 0 |  |  |  |  |
| Female | -0.02 | 0.03 | -0.58 | 0.56 | (-0.07;0.04) |
| **Caregiver’s sex** |  |  |  |  |  |
| Male | 0 |  |  |  |  |
| Female | 0.07 | 0.05 | 1.41 | 0.16 | (-0.03;0.17) |
| **IQ child^2^** | 0.005 | 0.002 | 3.61 | **<0.001** | (0.002;0.008) |
| **IQ caregiver**^2^ | 0.002 | 0.002 | 1.09 | 0.28 | (-0.002;0.006) |
| **Caregiver single** | 0 |  |  |  |  |
| **Caregiver not single** | 0.07 | 0.03 | 2.15 | **0.03** | (0.01;0.14) |
| **FHR**^3^ |  |  |  |  |  |
| FHR_SZ | -0.08 | 0.04 | -2.10 | **0.04** | (-0.15;-0.01) |
| FHR_BP | 0.04 | 0.04 | 0.91 | 0.36 | (-0.04;0.11) |
| PBC | 0 |  |  |  |  |
| **Caregiver unemployed** | -0.01 | 0.05 | -0.24 | 0.81 | (-0.10;0.08) |
| **Caregiver employed** | 0 |  |  |  |  |
| **Severe life events 0-3 years old** |  |  |  |  |  |
| 0 | 0 |  |  |  |  |
| 1 | 0.01 | 0.03 | 0.36 | 0.72 | (-0.06;0.08) |
| 2 | -0.03 | 0.04 | -0.66 | 0.51 | (-0.11;0.06) |
| 3 | -0.07 | 0.07 | -1.04 | 0.30 | (-0.21;0.06) |
| **Severe life events 4-7 years old** |  |  |  |  |  |
| 0 | 0 |  |  |  |  |
| 1 | -0.003 | 0.04 | -0.09 | 0.93 | (-0.07;0.07) |
| 2 | -0.03 | 0.04 | -0.78 | 0.44 | (-0.11;0.05) |
| 3 | -0.06 | 0.05 | -1.06 | 0.29 | (-0.17;0.05) |
| **K-SADS**^4^ **present diagnosis** |  |  |  |  |  |
| No | 0 |  |  |  |  |
| Yes | -0.02 | 0.03 | -0.58 | 0.56 | (-0.08;0.04) |
| **Diagnosis of substance use of primary caregiver** |  |  |  |  |  |
| No | 0 |  |  |  |  |
| Yes | 0.07 | 0.07 | 1.07 | 0.28 | (-0.06;0.21) |
| **PSP primary caregiver** | 0.006 | 0.002 | 3.75 | **<0.001** | (0.003;0.009) |
|  | F(18,444)=6.68; p<0.0001; R^2^=0.21; Adj. R^2^=0.18; Root MSE= 0.30 | | | | |

^1^In the case of siblings; parent and child information is only included from the first included sibling in order not to count the same parent twice. ^2^RIST, Reynolds Intellectual Screening Test. ^3^Familiar High Risk status (SZ: Schizophrenia, BP: Bipolar disorder, PBC: population-based controls). ^4^K-SADS, Kiddie Schedule for Affective Disorders and Schizophrenia.
